# Qi-Deficiency Related Increases in Disease Susceptibility Are Potentially Mediated by the Intestinal Microbiota

**DOI:** 10.1155/2018/1304397

**Published:** 2018-10-23

**Authors:** Ke Ma, Jieyu Chen, Liuyan Kuang, Jianlu Bi, Jingru Cheng, Fei Li, Xiaomin Sun, Xiaoli Nie, Yanyan Liu, Ren Luo, Xiaoshan Zhao

**Affiliations:** ^1^School of Traditional Chinese Medicine, Southern Medical University, Guangzhou, Guangdong 510515, China; ^2^Department of Endocrinology, Guangdong Second Traditional Chinese Medicine Hospital, Guangzhou 510095, China; ^3^Department of Nephrology, The First Affiliated Hospital of Zhengzhou University, Zhengzhou 450002, China; ^4^Department of Traditional Chinese Medicine, The Affiliated Ganzhou Hospital of Nanchang University, Ganzhou 314000, China

## Abstract

Qi-deficiency (QX) is thought to promote the body's susceptibility to disease, but the underlying mechanism through which this occurs is not clear. We surveyed the traditional Chinese medicine constitution (TCMC) of healthy college students to identify those that were PH (balanced TCMC constitution) and QX (unbalanced TCMC constitution). We then used high-throughput sequencing of the 16SrRNA V3-4 region in fecal microbiota samples to identify differences between those obtained from PH and QX individuals. Our results demonstrated that the alpha diversity of QX samples was significantly lower than that of PH samples (*p *< 0.05) and that beta diversity was remarkably different in QX and PH samples. Four and 122 bacterial taxa were significantly overrepresented in QX and PH groups, respectively. The genera* Sphingobium*,* Clostridium,* and* Comamonas* were enriched in the QX group and had a certain pathogenic role. The QX group also showed a statistically significant lack of probiotics and anti-inflammatory bacteria such as* Bifidobacterium *and* Bdellovibrio*. The functional potential of QX bacterial taxa was reduced in fatty acid metabolism and butanoate metabolism. We contend that the imbalanced intestinal microbiota in QX and the following functional changes in metabolism influence immunity and energy metabolism, which could increase susceptibility to disease.

## 1. Introduction

Traditional Chinese medicine constitution (TCMC) refers to relatively stable physical and psychological characteristics and is based on epidemiological investigations [1], clinical manifestations [2], and genomic research among Han Chinese [3, 4]. There is one balanced constitution of TCMC, PH, and eight unbalanced constitutions, including Qi-deficiency (QX) [5]. TCMC theory digitizes empirical Traditional Chinese Medicine diagnosis and treatment and provides a standardized guideline which is particularly useful for clinics [6]. Previous findings show that QX is an early stage in cancer [7, 8], heart disease [9], hypertension [10], diabetes [11], chronic fatigue syndrome [12, 13], and depression [14] and that PH is a protective factor against these conditions [9, 15].

PH people are energetic and fit, are not susceptible to illness, and have stable and powerful pulses and quality sleep. QX people are characterized by fatigue, are lacking in strength, and have weak pulses. QX is related to overwork, working pressure, and aging [16]. QX could promote the body's susceptibility to disease, but its mechanism is not clear.

Studies have shown the downregulation of immune-related genes including those involved in major histocompatibility complex [17] and interleukin-1*β* binding [18] in QX people. Our previous studies revealed the presence of metabolic disorders in the plasma of healthy QX people, including the presence of betaine and alanine [19]. Previous reports indicated that betaine in plasma positively correlates with* Clostridium* in feces [20] and that* Lactobacillus casei Shirota* could improve plasma alanine-aminotransferase levels [21]. These cases revealed that the specific plasma metabolites of QX were associated with intestinal microbiota. However, visceral manifestation of QX has also indicated spleen- and lung-deficiencies and some results indicate that these deficiencies induce compositional and functional changes in intestinal microbiota [22, 23].

We hypothesize that QX-related increases in disease susceptibility are potentially mediated by intestinal microbiota. We performed pyrosequencing of the 16SrRNA gene in healthy QX and PH Han Chinese college students, to compare their intestinal microbiota, and predicted metabolic function to explore the biological mechanism of QX.

## 2. Method

### 2.1. Study Subjects

**Figure 1:**
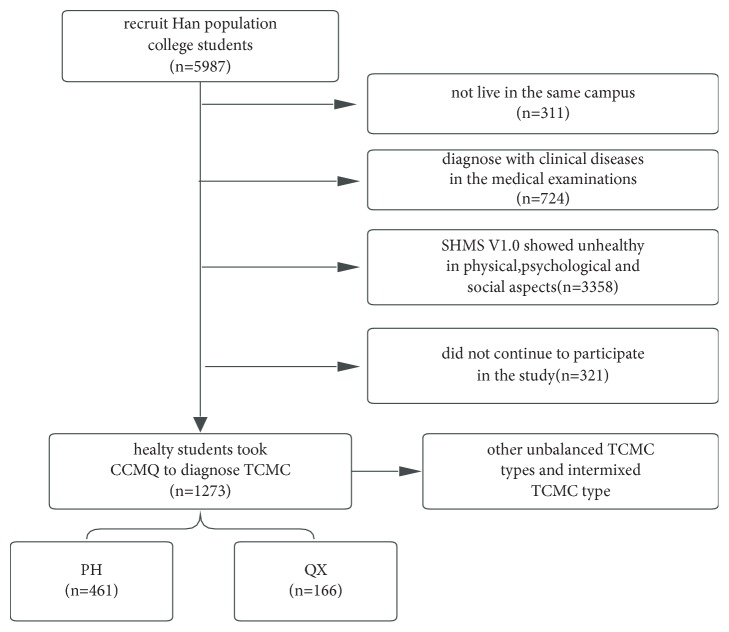
The sequence flow diagram of inclusion criteria for PH samples and QX samples.

This study is reviewed and approved by China Ethics Committee of Registering Clinical Trials (No. ChiECRCT-20170064) and Chinese Clinical Trials Registry (No. ChiCTR-IOR-17012986). We screened 461 healthy PH students and 166 QX students from 5987 college students of southern Han ethnicity (Figure 1). The inclusion criteria were as follows: first, unremarkable physical and blood tests including urea, electrolytes, and liver function tests; second, healthy status according to the results of Subhealth Measurement Scale V1.0 [24]; third, results of PH/QX in Chinese Constitution Medicine Questionnaire [25, 26]. Random sampling was used to select 21 PH and 21 QX students for 16SrRNA sequencing (V3–4). These 42 students were prohibited to take antibiotics, probiotics, or Chinese herbal medicines for 16 weeks. We obtained 16 PH and 19 QX sequencing datasets.

### 2.2. Fecal Sample Collection and Sequencing

Fresh fecal samples were collected in sterile container, kept at 4°C (<0.5 h), and then frozen at −80°C. According to the manufacturer's instructions, total fecal DNA was extracted using DNA Stool mini kit (Shenzhen Bioeasy Biotechnologies, Co., Ltd.). We amplified the V3-4 region of the 16SrRNA gene by PCR using the universal primers V4F and V4R (Table S1). 25 *μ*l reaction mixture was 0.25*μ*l 5U/*μ*l Premix Taq, 1 *μ*l template DNA, 0.5 *μ*l 10 *μ*M barcodes forward primer, 0.5 *μ*l 10 *μ*M reverse primer, and 16.75 *μ*l double-distilled H_2_O. The PCR cycle conditions were an initial denaturation at 94°C for 2 min, 30 cycles of 94°C for 30 s, 52°C for 30 s, and 72°C for 40 s, and a final extension at 72°C for 5 min. PCR products were sequenced using Illumina MiSeq instrument (PE 300, Illumina, San Diego, California, USA) at Guangzhou RiboBio Co., Ltd.

Sequencing reads were clustered by Illumina paired barcoded-sequencing (end) (BIPES) (PE) process for preliminary analysis; the rest of the sequence was screened by UCHIME and the suspected chimeric sequence was removed. The two-stage clustering (TSC) was used for extracting the OTU, with sequences having greater than or equal to 97% similarity being assigned to the same OTU. Alpha diversity was performed with Chao1, observed species, Shannon and Simpson. Beta diversity was performed with principle coordinate analysis (PCoA) based on UniFrac distance [27]. The linear discriminant analysis (LDA) with effect size measurements (LefSe) [28] was used to identify statistically significant different bacterial species in QX group and PH group. PICRUSt [29] was used to predict the metabolic function of the 16SrRNA gene datasets with KEGG Orthologs classification (*p*<0.05, Welch's t-test) [30].

### 2.3. Statistical Analysis

Epidata 3.0 was used to establish a database. Enumeration data was tested by chi-square. quantitative data was tested by independent-samples t-test. Kruskal-Wallis, PCoA, LEfSe, and PICRUSt analyzed the sequencing data. Statistical analyses were performed with the SPSS 20.0 (SPSS Inc., Chicago, IL, USA). P < 0.05 was considered to be of statistical significance.

## 3. Results

### 3.1. Students and Samples

There was no statistical significance between the two groups in age, sex, BMI, and health status scores (Table 1,* p*>0.05). We obtained 119,909 sequences from 19 QX samples and 218,480 sequences from 16 PH samples (Table S2).

### 3.2. Microbiota in QX Were Different from PH

**Figure 2:**
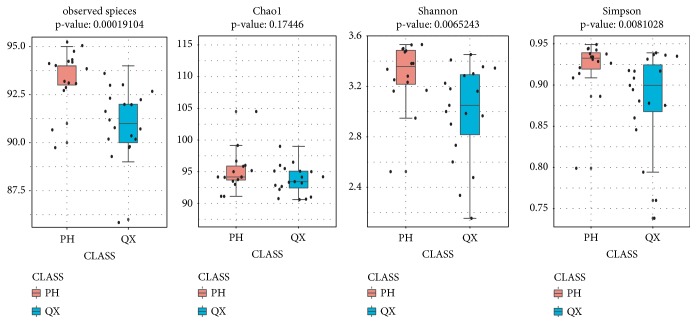
Bacterial alpha diversity in QX (blue) and PH (red) populations. Observed species, Shannon and Simpson indices demonstrate that the diversity in QX people is significantly lower than that in PH people. Mann-Whitney,* p* < 0.01.

**Figure 3:**
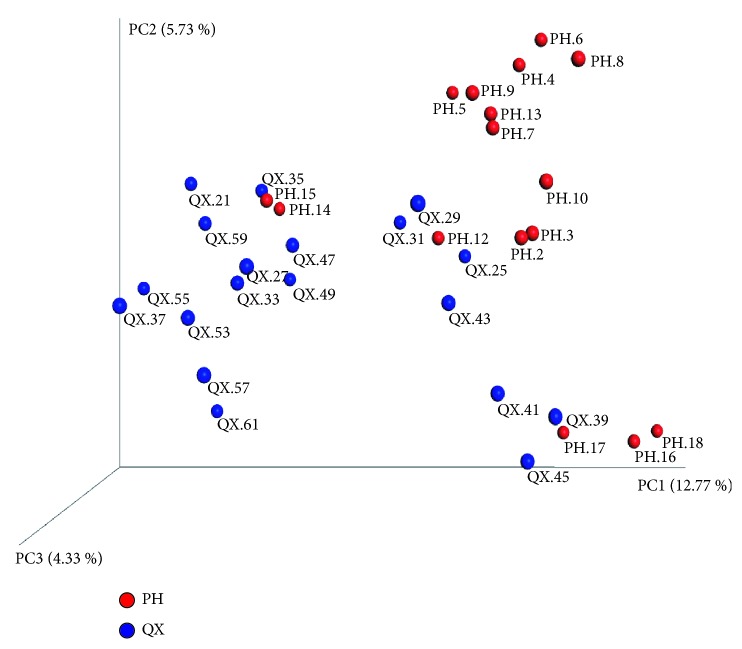
PCoA (unweighted) of intestinal microbiota in QX (blue) and PH (red) populations. Principal coordinate axis 1 (12.99% variability) and principal coordinate axis 2 (5.68% variability) highlight a clear clustering.

The numbers of observed species, Shannon and Simpson indices, were significantly lower in the QX group than in the PH group (*p*<0.05, Figure 2). PCoA (UniFrac) demonstrated a clear clustering of the microbial populations of the QX group, which were separated from the PH group (Figure 3).

### 3.3. Phylum, Family, and Genus Level Taxonomic Distribution of Intestinal Microbiota in QX Populations

**Figure 4:**
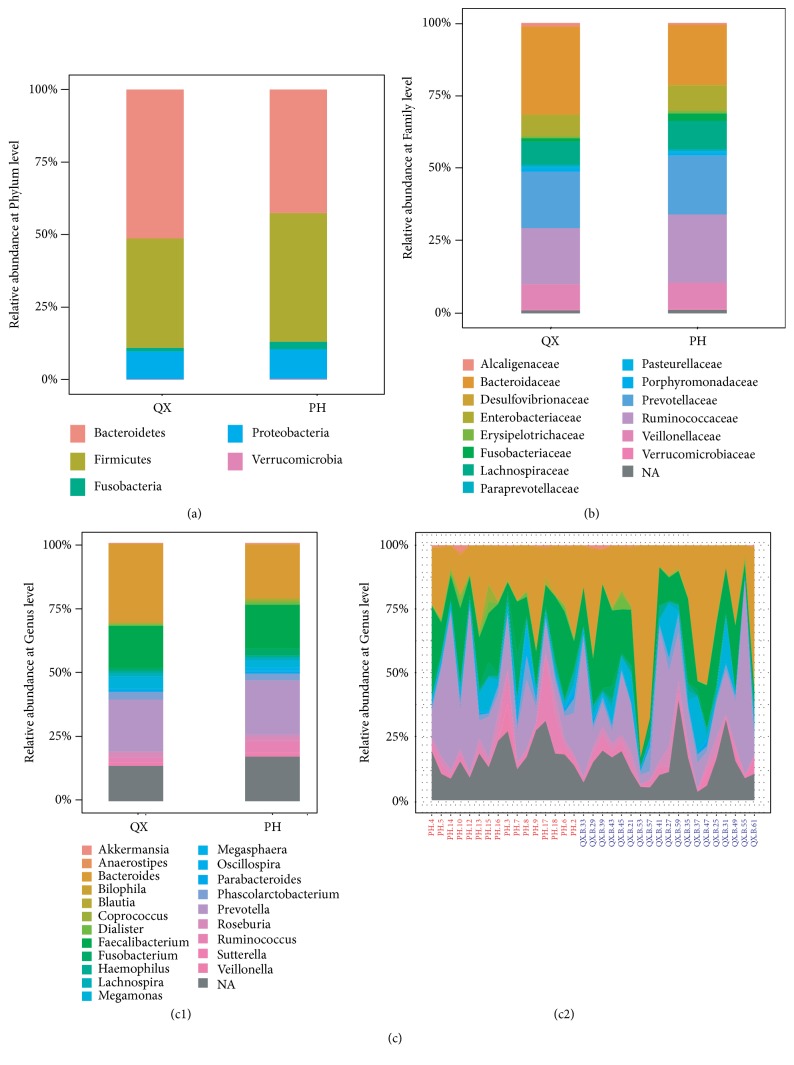
Relative abundance of intestinal microbiota at phylum (a), family (b), and genus (c) levels in PH and QX populations based on 16SrRNA sequencing. (b) shows that the taxa median relative abundance at the family level is ≥ 1% of total abundance. (c) shows that the taxa relative abundance at the genus level is ≥ 1% of the total abundance within groups (c1) and individuals (c2).

*Bacteroidetes* and* Firmicutes* were the top two phyla identified in QX samples and accounted for 47.64% and 39.23% of the total valid reads, respectively. In PH samples,* Firmicutes *and* Bacteroidetes* accounted for 43.99% and 38.52% (Figure 4(a)). At the family level, based on the average relative abundance, nine species were dominant (≥1%) in PH samples, and 10 species were dominant in QX samples. Bacteroidaceae and Alcaligenaceae were enriched in QX, whereas Ruminococcaceae, Fusobacteriaceae, and Bifidobacteriaceae were enriched in PH (Figure 4(b)). At the genera level, 12 genera were dominant in PH, and 11 genera were dominant in QX.* Bacteroides*,* Megamonas*,* Lachnospira*, and* Sutterella *were enriched in QX, and* Ruminococcus*,* Fusobacterium*,* Megasphaera*, and* Coprococcus* were more abundant in PH (Figure 4(c)). PH contained more* Ruminococcus* and* Coprococcus* which can produce butyrate [31], whereas* Sutterella*, present in QX samples, could give rise to unbalanced microflora by degrading secretory IgA and IgA itself [32].

### 3.4. Decreased Bacteria Associated with Short-Chain Fatty Acid Production and Butanoate Metabolism Are Prominent Features of QX

**Figure 5:**
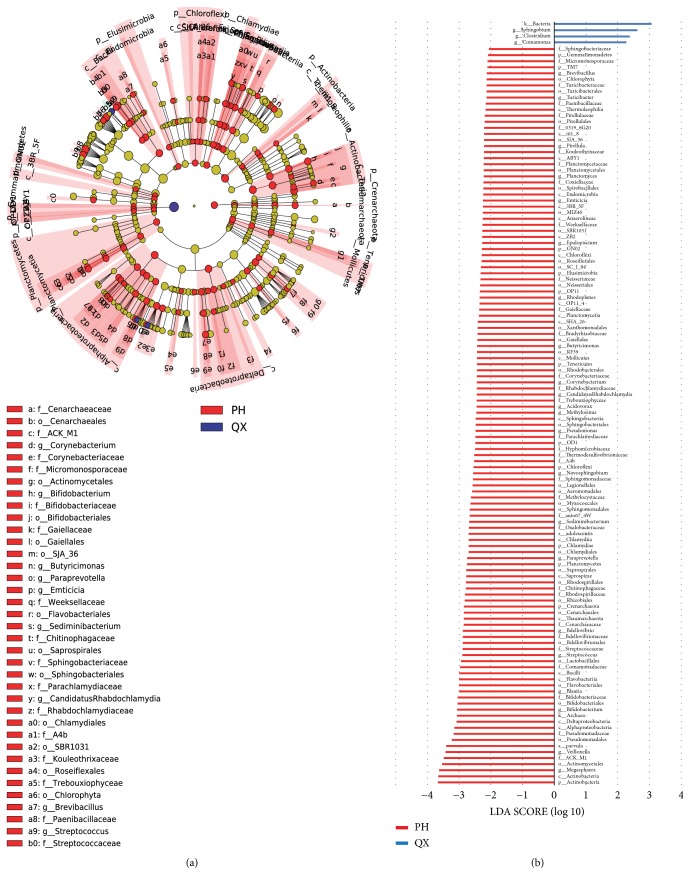
LefSe shows significantly discriminative features in QX (blue) and PH (red) populations. LDA > 2.0, p < 0.05. (a) Cladograms of the hierarchical structure. (b) Differential abundance bacterial taxa between the two groups presented as a histogram. The key microbiota taxa in QX were genus* Sphingobium, Clostridium,* and* Comamonas*.

LefSe showed biomarkers for QX and PH (LDA>2,* p*<0.05). Four and 122 taxa were enriched in QX and PH samples, respectively (Figures 5(a) and 5(b)). At the genus level,* Sphingobium*,* Clostridium*, and* Comamonas *were significantly overrepresented in QX and* Megasphaera*,* Veillonella*,* Veillonella parvula*,* Bifidobacterium*,* Blautia*,* Streptococcus*,* Bdellovibrio*, and* Paraprevotella* were significantly overrepresented in PH. Moreover, at the family level, Veillonellaceae, Bifidobacteriaceae, Pseudomonadaceae, Lactobacillales, Lachnospiraceae, Streptococcaceae, and Bdellovibrionaceae were significantly overrepresented in PH.

Of the biomarkers for QX,* Comamonas* is a cellulolytic microbe [33] and* Sphingobium *[34] and* Clostridium *[35, 36] are associated with inflammation. Of the biomarkers PH for,* Megasphaera *[37],* Veillonellaceae *[38],* Pseudomonadaceae* [39],* Streptococcaceae *[40], and* Bifidobacterium *[41, 42] can produce and metabolize fat, starches, protein, and polysaccharides, which are significantly lacking in QX. PH biomarkers* Bifidobacterium* and* lactobacilli* can ferment inulin-type fructans to cross-feed the microbiota and significantly stimulate the production of n-butyrate [43, 44]. Additionally, PH samples were rich in anti-inflammatory bacteria, including* Bifidobacterium *[45, 46],* Streptococcus *[47],* Lachnospiraceae *[48], and* Bdellovibrio *[49]. Metabolites of* Bifidobacterium,* including acetic acid, lactic acid, ethanol, formic acid, vitamins, and amino acids, can also stimulate the immune response and enhance the function of NK cells and the proliferation of T lymphocytes[50, 51].* Bdellovibrio*, considered as potential antibiotic substitutes, could also increase* Coprococcus*, which is reduced in irritable bowel syndrome and influences microbial balance [52].

**Figure 6:**
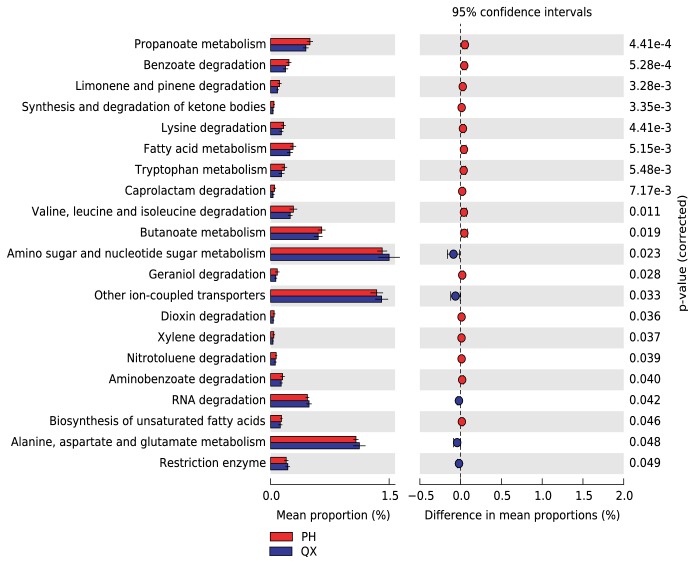
PICRUSt shows that the 21 predicted KOs significantly differ in their distribution in healthy QX (blue) and PH (red) populations. Functional modules involved in amino and nucleotide sugar metabolism and in RNA degradation are more abundant in QX than they are in PH. P < 0.05, Welch's t-test, Welch's inverted CI method, and effect size > 0.01.

PICRUSt was used to investigate the potential function and metabolic pathways of the taxa, based on Kyoto Encyclopedia of Genes and Genomes (KEGG) modules. Of note, QX had a lower proportion of bacterial genes related to fatty acid metabolism and butanoate metabolism and a higher proportion of bacterial genes related to amino and nucleotide sugar metabolism and RNA degradation (Figure 6,* p*<0.05, Welch's inverted CI method effect size>0.01).

## 4. Discussion

Here, we described our investigation of intestinal microbiota characteristics among healthy QX and PH Han college students in Southern China. We performed 16SrRNA gene sequencing to examine the different microbiota in QX and PH groups and to explain the biological mechanism underlying susceptibility to disease in QX individuals. Our results show that the bacterial taxa data of healthy people were similar to that of Asian populations reported in previous studies [53, 54] but that the microbiota of QX group was statistically different from that of the PH group.

Functionally, QX microbiota have relatively decreased fatty acid and butanoate metabolism potentials and relatively enriched carbohydrate metabolism (amino sugar and nucleotide sugar metabolism) and RNA degradation potentials. The imbalance of energy metabolism caused by alterations in intestinal microbiota is associated with QX-related symptoms. QX people demonstrate significantly higher BMI scores and waist-to-hip ratios and significantly lower scores in back muscle strength [55]. QX symptoms include a reduction of resting metabolic rate, which results in flabby muscles, vulnerability to fatigue, feeble pulse, shallow breathing [56], and potential concern with disturbances in energy metabolism. Research has shown that GLUT4, a critical protein in glucose metabolism, increases following low-intensity exercise [57] and that moderate-intensity exercise increases fatty acid oxidation to adapt energy metabolism [58]. Per mole of oxygen consumed, the oxidation of carbohydrates produces 15% more ATP than the oxidation of lipids does [59]. However, lipid oxidation produces greater energy density compared to carbohydrate oxidation, and fatty acids can go straight to the muscles' mitochondria for energy. Therefore, we postulate that QX and PH people adopt different optimal-fuel strategies to acclimatize themselves to daily energy expenditure. Compared with PH people, QX people tend to adopt a carbohydrate-based metabolism to prolong the time for which energy is maintained, which makes it possible to induce the symptoms of lower metabolic rate. PH people tend to adopt fatty acid metabolism and butanoate metabolism. Lipid oxidation has a greater energy density than does carbohydrate oxidation, which efficiently elevates thermogenesis capacity. Higher rates of fat oxidation generally reflect well-trained body conditions, while lower fat oxidation rates may be related to obesity and insulin resistance [60].

The microbiota identified in the QX group had reduced levels of probiotics and decreased anti-inflammatory bacteria than did those identified in the PH group. The alteration of intestinal microbiota and unbalanced metabolism in QX, including reduction of fatty acid and butanoate metabolism, can increase disease susceptibility. Short-chain fatty acids (SCFAs), especially butyrate, play an important role in the regulation of host immunity. SCFAs directly contact intestinal epithelium cells (IECs), and SCFAs, especially butyrate, are used as ATP sources for energy metabolism. However, SCFAs also enhance IEC immune surveillance by increasing the expression of certain antimicrobial peptides and maintaining the integrity of intestinal mucosa [61–63]. For example, commensal microbe-derived butyrate induced the differentiation of colonic regulatory T cells, which have a central role in the suppression of inflammatory and allergic responses [64]. Furthermore, butyrate and valproic acid upregulated B cell microRNAs in human and mouse B cells to modulate antibody and autoantibody responses [65].

In conclusion, this study demonstrates that intestinal microbiota in the QX group showed lower biological diversity and possessed a different microbial signature than did that of the PH group. We contend that the QX's imbalanced intestinal microbiota and concomitant functional changes in metabolism potentially influence immunity and energy metabolism, which could increase susceptibility to disease.

## Figures and Tables

**Table 1:** General situation.

characteristics	QX (n = 19)	PH (n= 16)	*p* value
Age (years)	20.75 ±1.47	20.13±1.31	0.2007
Sex (male /female)	7/12	5/11	0.728
BMI (kg/m^2^)	20.18±3.31	20.94±1.31	0.3951
QX transformed score	48.58±9.56	10.94±5.82	—
Health Status Scores	79.23±5.32	81.47±7.15	0.2962

No statistical significance between the two groups in age, sex, BMI, and health status scores (*p*>0.05).

## Data Availability

The data used to support the findings of this study are available from the corresponding author upon request.

## Supplementary Materials

Supplemental Materials Table S1. The information of primers. Table S2. Samples sequencing and alpha diversity of the intestinal microbiota.
